# Sedentary Behaviour and Hair Cortisol Amongst Women Living in Socioeconomically Disadvantaged Neighbourhoods: A Cross-Sectional Study

**DOI:** 10.3390/ijerph15040586

**Published:** 2018-03-25

**Authors:** Megan Teychenne, Dana Lee Olstad, Anne I. Turner, Sarah A. Costigan, Kylie Ball

**Affiliations:** 1 Institute for Physical Activity and Nutrition (IPAN), School of Exercise and Nutrition Sciences, Deakin University, Geelong 3220, Australia; dana.olstad@ucalgary.ca (D.L.O.); anne.turner@deakin.edu.au (A.I.T.); kball@deakin.edu.au (K.B.); 2 Department of Community Health Sciences, Cumming School of Medicine, University of Calgary, Calgary T2N 4Z6, AB, Canada; 3 School of Exercise and Nutrition Sciences, Deakin University, 221 Burwood Hwy, Burwood, VIC, 3125, Australia; sarah.costigan@deakin.edu.au

**Keywords:** sitting, screen-time, psychological stress, socioeconomic disadvantage

## Abstract

Women living in socioeconomically disadvantaged neighbourhoods are at heightened risk of experiencing psychological stress. Therefore, identifying potential risk factors for stress is important to support positive mental health. A growing body of research has linked sedentary behaviour with mental ill-health (e.g., depression and anxiety); however, little research has specifically investigated potential linkages between sedentary behaviour and stress. Therefore, the aim of this study was to investigate the association between common types of sedentary behaviour and objectively-measured stress (as measured by hair cortisol levels) amongst women living in socioeconomically disadvantaged neighbourhoods. During 2012–2013, 72 women (aged 18–46 years) living in socioeconomically disadvantaged neighbourhoods self-reported sedentary behaviour (TV viewing, computer use, overall sitting time) and provided hair samples. Hair cortisol levels were measured using enzyme-linked immunosorbent assay. Linear regression models examined cross-sectional associations between sedentary behaviour and hair cortisol levels. There was no association between any type of sedentary behaviour (TV viewing, computer use, or overall sitting time) and hair cortisol levels in either crude or adjusted models. Sedentary behaviour may not be linked to hair cortisol level (stress) in women living in socioeconomically disadvantaged neighbourhoods. Further studies utilising objective measures of both sedentary behaviour and stress are required to confirm these findings.

## 1. Introduction

High levels of psychological stress are linked to poor mental and physical health outcomes [1]. It has been suggested that 75–90% of visits to general practitioners in the U.S. are related to stress, with direct and indirect costs totalling hundreds of billions of dollars annually [2]. Therefore, it is essential to explore approaches that may prevent and/or manage stress in the population. 

A growing body of research has linked sedentary behaviour (i.e., sitting or reclining behaviours that expend minimal energy such as television (TV) viewing, sitting at the computer) with poor mental health, and in particular with anxiety [3] and depressive symptoms [4]. Given the possible adverse effect of sedentary behaviour on those mental health outcomes, one could expect sedentary behaviour to also increase level of stress. This is particularly suggested since activities that may theoretically induce stress, such as working on a computer or using electronic devices for purposes including reading the news or engaging with social media, are often undertaken whilst sitting. However, very little research has examined associations between sedentary behaviour and stress, and the limited existing evidence has been inconsistent. For example, in studies that have utilised subjective measures of stress, one prospective study showed that perceived stress was associated with medium levels of television viewing (14–21 h per week) but not overall sitting time, amongst women living in socioeconomically disadvantaged neighbourhoods [5]. In cross-sectional studies, feeling somewhat stressed was associated with higher sitting time in one study of young women [6], while in another study time spent sitting for transport was linked to higher perceived stress in men and women, yet sitting at the computer, watching TV, and work-related and leisure-sitting were not [7]. It is possible that these mixed findings may partly reflect the use of subjective measures of stress which may be subject to social desirability bias, whereby participants are likely to under-report stress or other indicators of adverse mental states [8]. Using objectively-measured indicators of stress might therefore help to minimise some forms of measurement error inherent within subjective tools. 

To date, few studies have examined the association between sedentary behaviour and objectively-measured indicators of stress (i.e., using salivary, plasma, urinary, or hair cortisol). Cross-sectional research has shown that TV viewing was associated with lower salivary cortisol levels (as measured during late afternoon/early evening) in women and that playing video games was associated with higher salivary cortisol levels in the whole sample [9]. However, most of the studies that have examined associations between sedentary behaviour and objectively-measured indicators of stressful exposures were conducted in adolescent populations (specifically boys) and assessed video game playing. Such studies cannot show how other more common sedentary behaviours such as TV viewing, computer use, and overall sitting might relate to stress. Further, most used salivary cortisol to measure stress, which is difficult to interpret given substantial diurnal fluctuations. Hair cortisol levels, by contrast, reflect cortisol exposure over several months [10]. Women, in particular those living in socioeconomically disadvantaged neighbourhoods, are more likely to experience psychological stress compared to men [11] or those living in more advantaged neighbourhoods [12]. Socioeconomically disadvantaged women may face a number of issues including unemployment, financial insecurity, and living in neighbourhoods characterised by higher crime rates, safety concerns, and visible signs of disorder, all of which may be potential sources of stress. Therefore, it is important that research targets this population in order to identify potential risk factors for, and/or behavioural outcomes of, stress. Thus, the aim of this study was to investigate the association between common types of sedentary behaviour and an objectively measured indicator of stress (hair cortisol) amongst women living in socioeconomically disadvantaged neighbourhoods.

Given that sedentary behaviour (in particular overall sitting and computer use) has been linked to higher risk of mental health outcomes such as anxiety and depression [4,13], it was hypothesised that higher levels of sedentary behaviour would be linked to higher levels of stress, with high levels of stress associated with clinical conditions such as depression and anxiety in susceptible individuals [14]. Although mechanisms are not well understood, sedentary behaviour could potentially cause stress via a number of pathways. A large proportion of the population utilise social media platforms when engaged in sedentary behaviour (e.g., sitting/computer use). Excessive use of social media has been shown to lead to feelings of addiction and loss of sleep amongst women [15], which could subsequently induce stress. Alternatively, it has been hypothesised that spending time using computers (or electronic devices) can disengage women from other responsibilities (e.g., household chores, engaging with their children) which could enhance feelings of anxiety and stress [13]. Sedentary behaviour may displace time spent exercising, which is known to have mental health benefits, including reduction in stress [16]. Finally, the relationship with sedentary behavior may operate in the reverse direction; that is, highly stressed women may choose to sit more (e.g., watch TV) as a way to destress [17].

## 2. Materials and Methods 

Data from the third wave of the Resilience for Eating and Activity Despite Inequality (READI) prospective cohort study (2012–2013) were used in this cross-sectional analysis. Ethical approval was granted by the Deakin University Human Research Ethics Committee (HEAG-H 91_2006) and written consent was obtained from all participants. Study methods have been described in detail elsewhere [18,19] and are briefly outlined below. 

### 2.1. Participants and Procedures

Based on the Australian Bureau of Statistics Socioeconomic Index for Areas (SEIFA), 80 socioeconomically disadvantaged Victorian suburbs (40 urban and 40 rural suburbs selected randomly from the lowest tertile of neighbourhood disadvantage) were nominated as READI study neighbourhoods. Up to 150 women (aged 18–46 years) from each of those neighbourhoods were randomly selected using the electoral roll and received a written, mailed invitation to participate in the study. In 2007–2008 (Wave 1), 11,940 women were mailed surveys and of those 4934 (41%) returned completed surveys (eligible women for READI study = 4349). At Wave 2 (2010–2011), 1913 of those women completed a follow-up survey, and at Wave 3 (2012–2013), 1560 women completed follow-up surveys. Of Wave 3 participants, 199 women were randomly selected and invited to participate in a sub-study involving hair cortisol analysis. A further 266 women who had completed both a mother and child survey (i.e., women with children under the age of 12 years at baseline who had participated in the READI child sub-study, *n* = 613 at T1, *n* = 382 at T2, *n* = 277 at T3) at Wave 3 and who had consented to further follow-up were also invited to participate. Across these two samples, a total of 116 women agreed to participate in the hair cortisol sub-study. Pregnant women were excluded.

### 2.2. Measures

Weekly sedentary behaviour was assessed using three measures: time spent sitting while watching TV, time spent sitting while using the computer, and overall sitting time. Time spent watching TV and using the computer were separately assessed using reliable measures [20,21], whereby participants estimated the hours and minutes they spent engaged in those activities on a usual weekday and weekend day in the past week. The duration of sitting on weekdays (i.e., single day estimate multiplied by five) and weekend days (i.e., single day estimate multiplied by two) were added together to derive a weekly total for time spent sitting while watching TV and a separate weekly total of time spent sitting while using a computer. For overall sitting time, participants estimated the hours and minutes they spent sitting on a usual weekday and weekend day in the past week [22] and weekly totals were calculated as above.

Hair cortisol analysis was used as an objective indicator of levels of stress over the past 1–3 months. A hair sampling kit with instructions was sent to consenting participants. Women cut approximately 100 strands of their hair (as close to scalp as possible) from the posterior vertex location on the head. Women marked the scalp end of the hair and taped the sample to a paper slip provided and returned this to the research team by mail in a sealed envelope. Within 3–4 months of survey completion, 102 hair samples were received (22% of invited participants). Of those, 28 were excluded due to having non-usable samples (e.g., too few or short strands, did not mark scalp end), which left a total of 74 women with data for inclusion. Details of the hair sample preparation and cortisol extraction procedures are outlined elsewhere [18]. Cortisol levels were measured using an Enzyme-Linked Immunosorbent Assay for salivary cortisol (Salivary ELISA Cortisol kit; Alpco Diagnostics, Windham, NH, USA), a reliable and valid method of measuring cortisol [23]. Analysis was performed on the average cortisol levels across the three hair segments where available (93% of women), otherwise one or two segments were used (7% of women) [24]. 

### 2.3. Analyses

Of the 74 women with hair cortisol data, two were excluded as they had extreme outlier values for average cortisol level (>3 SD above the mean), leaving 72 participants with data for inclusion in analyses. The outcome variable (hair cortisol level) was tested for normality and subsequently transformed by natural log as it showed violations of the normality of residuals and homoscedasticity assumptions. We used log-transformation of hair cortisol level (outcome variable), which was found to better meet these model assumptions. Crude and adjusted linear regression models were used to test associations between sedentary behaviour variables (TV viewing, computer use, overall sitting time) and hair cortisol level. Models were adjusted for self-reported level of education (did not complete tertiary education, completed tertiary education), self-reported leisure-time physical activity (hours/week) using items from the reliable and valid International Physical Activity Questionnaire [22], and hair dying (self-reported yes vs. no), since these variables were theoretically linked to the outcome variable [25]. Just one exposure variable had missing data (sitting time, *n* = 1) and thus that one case was excluded from analyses where sitting time was the exposure variable.

## 3. Results

Table 1 presents the sociodemographic characteristics of participants. Most were married or in a de-facto relationship (~89%), had not completed tertiary education (~60%), had good to excellent self-rated health (~96%), and had children living at home (~79%). Hair cortisol concentration (HCC) ranged from 42.5 to 599 ng/g (mean 130.0 ng/g). There were no associations between any type of sedentary behaviour and cortisol levels in either crude or adjusted models (Table 2).

## 4. Discussion

This study showed that different types of sedentary behaviours (TV viewing, computer use) and overall sitting were not associated with hair cortisol level in women living in socioeconomically disadvantaged neighbourhoods. Previous studies have shown positive [9], negative [9], and null [26] associations between sedentary behaviours (namely video gaming and TV viewing) and cortisol level. Thus, further studies, in diverse population groups, are required to clarify these relationships. 

Women living in socioeconomically disadvantaged neighbourhoods are at heightened risk of experiencing stress [11,12]. Therefore, it may be that for women in this target group, engaging in sedentary behaviour, such as TV viewing and using computers, does not affect their stress levels, since they potentially have far greater stressors in their daily lives such as financial and/or neighbourhood safety concerns [27]. Acknowledging reverse directionality, another possible explanation for the null findings is that some women may utilise TV viewing, for example, as a coping strategy to reduce stress. Others may use other non-sedentary activities, such as exercise, as a coping strategy for when they are stressed, with these coping mechanisms potentially dependent on other factors such as personal interests and personality traits. It may be that studies examining the link between sedentary behaviour and stress need to also consider the particular coping strategies women use to deal with stress, which might differentially buffer the impact of stress. 

Limitations of this study included the cross-sectional design, the self-report measures of sedentary behaviour (which may be subject to recall difficulties and social desirability bias), and the low response rate, which may have limited the generalisability of results. Further, the small sample size may have limited the statistical power to detect associations and/or could present selection bias. As such, there were some key differences between the demographics of the larger READI sample and the sample in this sub-study. For example, the proportions of women with high (>AUS$1500/week) incomes (44.4% vs. 17.5%) or in paid employment (85% vs. 68%) were higher in the current subsample compared with the larger READI sample. Mean weekly time spent in TV viewing (20 h vs. 25 h), computer use (17 h vs. 22 h), and overall sitting (46 h vs. 51 h) was lower in the current subsample compared to the larger READI sample. Meanwhile, mean time spent in leisure-time physical activity was higher for the current subsample compared to the entire READI sample (by 1.2 h per week). Hence, findings may not be generalisable to the entire population of socioeconomically disadvantaged women. Survey questions related to computer use did not differentiate between work and leisure-time use and thus distinguishing between these two contexts may have yielded different results. Since the cortisol measure assessed cortisol levels over the previous 1–3 months, and the measure of sedentary behaviour assessed behaviours during the previous week, the mismatched time frame of measurement may have resulted in a weaker observed measure of association between these constructs than had they both been assessed over the same time frame. Mean (±SD) HCC was 130.0 ng/g (±89.8) in this study, which is higher compared with the HCC values reported by Manenschijn et al. [28] in a healthy population (mean HCC = 27.3 ng/g, 95% confidence interval = 24.6–30.4). Some of this difference is likely due to methodological differences between laboratories [29]. Further, since participants in the current study were living in socioeconomically disadvantaged neighbourhoods, it is possible that some of the high HCC in our sample may be attributed to chronic stress exposure associated with neighbourhood deprivation. Key strengths of this study included the assessment of common types of sedentary behaviours beyond video game use which are more relevant to adults. Further, we utilised an objective indicator of stress (hair cortisol), which is a useful measure for indicating cortisol exposure over time [10]. It should be acknowledged, however, that hair cortisol measures all exposure to cortisol and not just that which is induced by stress. For example, exogenous glucocorticoids which are used to treat conditions including chronic inflammatory or skin conditions may influence hair cortisol levels in a way that is not related to exposure to stress. However, participants were not asked about exposure to exogenous glucocorticoids. 

## 5. Conclusions

The current study showed no link between sedentary behaviour and hair cortisol level (stress) amongst women living in disadvantaged neighbourhoods. It is recommended that research studies with larger sample sizes, utilising objective measures of sedentary behaviour and stress, are further conducted in women in different socioeconomic contexts, in order to better understand factors that may influence, or be influenced by stress.

## Figures and Tables

**Table 1:** Sociodemographic characteristics of women (*n* = 72).

	Current Sub-Study (*n* = 72)		Entire Eligible Baseline READI Sample (*n* = 4349)	
**Characteristics**	***n***	**%**	***n***	**%**
**Smoking status**				
Never	46	63.9	2184	50.3
Former	22	30.6	1066	24.5
Current	4	5.5	1096	25.2
**Marital status**				
Married/de-facto	64	88.9	2829	65.5
Separated/divorced/widowed	8	11.1	1493	35.0
**Education**				
Did not complete tertiary education	43	59.7	3162	73.8
Completed tertiary education	29	40.3	1120	26.2
**Self-rated health**				
Poor to fair	3	4.2	633	14.6
Good to excellent	68	95.8	3699	85.4
**Children living at home?**				
Yes	57	79.2	2648	61.7
No	15	20.8	1642	38.3
**Employment status**				
Employed full time	31	43.0	1613	38.1
Employed part-time	30	42.0	1245	29.4
Not in paid employment	11	15.0	1372	32.4
**Country of birth**				
Australia	68	94.4	3851	88.9
Other	4	5.6	480	11.1
**Household income (AUD)**				
Low ($0–699/week)	7	9.7	1117	25.7
Medium ($700–1499/week)	23	31.9	1522	35.0
High (>$1500/week)	32	44.4	762	17.5
Not disclosed	9	12.5	948	21.8
**Hair dying**				
Yes	51	70.8	N/A	N/A
No	21	29.2	N/A	N/A
	**Mean**	**SD**	**Mean**	**SD**
Age	43.5	7.1	34.4	6.1
Body Mass Index (BMI)	26.3	5.9	26.1	6.1
TV viewing (hours/week)	20.4	21.7	25.2	25.1
Computer use (hours/week)	17.4	14.9	21.6	29.1
Overall sitting time (hours/week)	46.3	29.2	50.5	33.5
LTPA (hours/week)	4.6	5.9	3.4	4.6
Age	130.0	89.8		

LTPA, Leisure-time physical activity; READI, Resilience for Eating and Activity Despite Inequality.

**Table 2:** Linear regression analyses examining associations between sedentary behaviour and hair cortisol level (log-transformed).

Sedentary Behaviour (Hours/Week)	Crude Models				Adjusted Models *			
	B	95% CI	Exp(B) ^a^	*p* Value	B	95% CI	Exp(B) ^a^	*p* Value
TV viewing	−0.001	−0.007, 0.005	0.999	0.740	−0.002	−0.008, 0.005	0.998	0.612
Computer use	0.002	−0.007, 0.011	1.002	0.657	0.002	−0.007, 0.011	1.002	0.653
Overall sitting time	−0.001	−0.006, 0.004	0.999	0.659	−0.002	−0.007, 0.003	0.998	0.406

* Models adjusted for education level, leisure-time physical activity, and hair dying. ^a^ Exponentiated regression coefficient, representing proportionate increase in the geometric mean of the raw outcome variable per unit increase in the exposure.
